# An Abrasion, a Prosthetic Shoulder, and a Cat with a Licking Tendency: Case Report and Literature Review of *P. multocida* Joint Seeding

**DOI:** 10.1155/2020/2842315

**Published:** 2020-11-18

**Authors:** William F. Abel, Christopher S. Eckman, Robert P. Summers, William S. Sessions, Amanda E. Schnee

**Affiliations:** ^1^University of South Carolina School of Medicine Greenville, 607 Grove Rd., Greenville, SC 29605, USA; ^2^Prisma Health Upstate Department of Medicine, 701 Grove Rd., Greenville, SC 29605, USA; ^3^Prisma Health Upstate Department of Infectious Disease, 701 Grove Rd., Greenville, SC 29605, USA

## Abstract

*Pasteurella multocida* is a pathogen well known for its zoonotic transmission, most commonly by cats and dogs. When bacteremia ensures from an infection, patients with foreign objects present in their bodies, including prosthetic joints and mesh implants, become vulnerable to seeding. There have been multiple documented cases in which *P. multocida* bacteremia has resulted in infection of both native and prosthetic joints. Furthermore, cases have been documented in which patients with *P. multocida* bacteremia have developed meningitis and neurological complications. Here, we present a patient with multiple comorbidities including multifactorial immunocompromise, advanced age, and multiple prosthetic joints who developed prosthetic joint infection and spinal osteomyelitis after the development of *Pasteurella* bacteremia. Aggressive treatment was undertaken given her risk factors, and a combination of antibiotics and surgery was utilized, with the patient making a full recovery.

## 1. Background

*Pasteurella multocida* was first isolated by Louis Pasteur in 1880. Since that time, it has been demonstrated as a common source of zoonotic infection with *P. multocida* isolated in up to 50% of dog bites and 75% of cat bites [1]. *P. multocida* infection has also been documented in the absence of bite wounds in patients with cat scratches present on physical examination [2]. Less commonly still are instances in which *P. multocida* is isolated from an individual where no wounds from animals are detected [3, 4]. We present a case of *P. multocida* bacteremia in an immunocompromised patient, with a history of close contact with both cats and dogs but no bites or scratches, resulting in subsequent septic arthritis of the shoulder, epidural abscess, and spinal osteomyelitis. We reviewed the available literature on *P. multocida* and pathogenicity, antibiotic susceptibility profiles, and propensity for metastatic foci of infection.

## 2. Case Presentation

A 67-year-old female with a past medical history of multiple myeloma status postautologous stem cell transplant 4 years prior with evidence of stringent complete response and maintained on lenalidomide presented with wrist pain, fevers, and altered mental status. The patient had been well until two days prior when she fell at home. She was found by a home aide to be confused, febrile, and with a swollen right wrist. Significant surgical history included bilateral knee surgeries, left shoulder arthroplasty, and right total hip arthroplasty. She reported living with a roommate and numerous domestic cats and dogs.

In the emergency department, presenting vital signs revealed blood pressure 86/44 mmHg, temperature 101.1 F, heart rate 128, and oxygen saturation 94% on 3 liters nasal cannula. Examination of the patient revealed her to be alert and oriented only to herself. Respiratory examination revealed good air movement and normal breath sounds. The ulnar aspect of her right wrist revealed a small abrasion with mild erythema. The right wrist was edematous and tender to palpation. She had decreased strength and range of motion in the right wrist, but normal elbow and shoulder joints. There were no signs of otherwise broken or punctured skin. The remainder of her musculoskeletal examination was unremarkable.

Initial complete blood count revealed a white blood cell count of 5.0 Th/mm^3^ (normal limit 4.0–11.0 Th/mm^3^), hemoglobin 10.4 g/dL (grams/deciliter) (normal limit 12.0–16.0 g/dL), platelets 132.0 Th/mm^3^ (normal limit 150–400 Th/mm^3^), and 21% bands (normal limit 3–5%). Complete metabolic panel was significant for sodium 126 mMol/L (millimoles per liter) (normal limit 136–145 mMol/L), AST 213 IU/L (international units per liter) (normal limit 8–20 IU/L), ALT 57 IU/L (normal limit 8–20 IU/L), and alkaline phosphatase 479 IU/L (normal limit 20–70 IU/L). Erythrocyte sedimentation rate and C-reactive protein were 104 mm/hr (normal limit 0–20 mm/hr) and 237 mg/L (normal limit < 10 mg/L), respectively. Procalcitonin was 5.48 ng/mL (normal limit < 0.05 ng/mL). Urinalysis returned negative for leukocyte esterase and nitrite, and moderate blood was noted. Blood, urine, and stool cultures were obtained. Lumbar puncture was attempted twice unsuccessfully. Aspiration of the right wrist resulted in a dry tap. Empiric antibiotic coverage with vancomycin and ceftriaxone was initiated.  Table 1 illustrates lab values from the patient's admission.

**Figure 1:**
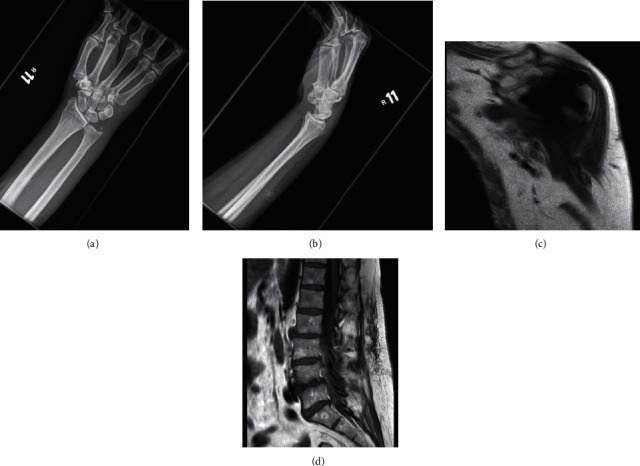
(a) PA X-ray right wrist, (b) lateral X-ray right wrist, (c) T1 coronal MRI right shoulder, and (d) T1 sagittal lumbar spine.

Radiographs of the chest as well as the right wrist (Figure 1), elbow, and humerus were obtained. Computed tomography (CT) of the head, abdomen, and pelvis were also obtained. Right upper extremity radiographs revealed no acute osseous or soft tissue abnormalities, and chest radiograph revealed clear lungs. CT imaging revealed no intracranial abnormalities but did note fluid levels in frothy secretions in the paranasal sinuses, as well as nonspecific perinephric edema and thickening of the renal pelvis bilaterally.

The patient's blood cultures grew *Pasteurella multocida* in both sets of bottles. Susceptibilities included amoxicillin-clavulanate, ampicillin, ceftriaxone, levofloxacin, penicillin, and tetracycline. The organism was noted to be resistant to trimethoprim-sulfamethoxazole. Respiratory, stool, and urine cultures were negative. Antibiotic therapy was deescalated to monotherapy with ceftriaxone. Repeat blood cultures were obtained on hospital day four and were without growth.

On hospital day seven, the patient reported worsening left shoulder pain, the site of her previous left total shoulder arthroplasty. Magnetic resonance imaging (MRI) (Figure 1) revealed the presence of a small perihardware collection. Aspiration was performed, revealing approximately 70,000 white blood cells without crystals, and no subsequent growth on either aerobic or anaerobic cultures. Arthroscopy revealed purulent drainage from the anterior left shoulder, and subsequent lavage was performed by orthopedics out of concern for septic arthritis. Thick, purulent material was noted intraoperatively, but cultures remained without growth.

On hospital day ten, the patient reported an acute worsening in pain and feeling overall unwell. Physical exam revealed extreme point tenderness to palpation of her cervical and lumbar spine. MRIs of the spine (Figure 1) revealed multilevel osteomyelitis and septic arthritis of the facet joints L3-S1 bilaterally, as well as edema of the paraspinous soft tissues with epidural abscess. Laminectomy with incision and drainage was performed, and subsequent cultures revealed no growth. Additional imaging with contrast further demonstrated septic arthritis and epidural abscess.

The patient was able to be successfully discharged to a rehabilitation facility with the follow-up arranged with infectious disease and orthopedic specialists. She was continued on levofloxacin at discharge to complete a total of 6 weeks of therapy.

## 3. Discussion

*P. multocida* is a Gram-negative organism found as a member of the normal flora in the oropharynx and nasopharynx of cats, dogs, cattle, and mice [5]. With 38.4% of households owning dogs and 25.4% of households owning cats in the United States, this is an organism that a relatively large proportion of the population is exposed to on a daily basis [6]. Of particular relevance is the danger of infection with *P. multocida* progressing to bacteremia, which has mortality rates up to 30% [7].

### 3.1. Virulence Factors

*P. multocida* possesses a number of virulence factors known to facilitate various diseases in both humans and animals. LPS (lipopolysaccharide) from *P. multocida* has been shown to cause hemorrhagic septicemia in ungulates, cholera in avian species, and atrophic rhinitis in pigs [8]. While there are no known exotoxins produced, the polysaccharide capsule is important for protection from host immune defenses, and the filamentous hemagglutinin surface adhesin allows for adherence to structures [8]. There are 5 capsular serogroups and 16 Heddleston serovars, with A:1 and A:3 (serogroup: serovar) being most commonly transmitted from cats to humans [9]. *P. multocida* possesses the ability to produce biofilms, although this ability depends on the conditions present and interferes with the production of serogroup A capsule [10]. It would seem plausible, then, that in an immunosuppressed host such as in our case, the need for capsule production to evade immune response would be lessened, allowing for the production of biofilms and potentially the ability to seed multiple sites within the same host. The ability to form biofilms in these hosts may also speak to the need for longer durations of therapy, more aggressive interventions where foreign bodies are involved, and the consideration for suppressive therapy in settings where foreign bodies cannot be removed. This hypothesis is substantiated by a case report by Guilbart et al. [11], where they report a patient who suffered fatal septicemia related to *P. multocida* cellulitis which was complicated by endocarditis as well as prosthetic joint infection. In this particular case, they attempted a salvage approach for the prosthetic joint but were unsuccessful at clearing the infection, indicating that the virulence factors discussed require more aggressive initial interventions for effective treatment.

### 3.2. Antibiotic Resistance

Drug resistance has been noted in a number of strains infecting bovine and avian species in China [12]. *P. multocida* strains known to infect humans and cats have been shown to possess resistance to cotrimoxazole (75.6%), sulfisoxazole (60.9%), and penicillin (7.3%), as well as others at very low rates [13]. Furthermore, *β*-lactamase production has been documented as well as the presence of plasmids that are associated with resistance to various antibiotic classes including tetracyclines and aminoglycosides [14]. The potential for beta-lactamase production should be noted by physicians when treating *P. multocida* infections or when *P. multocida* is suspected and treatment failure occurs, as beta-lactams are frequently the first line therapies for cellulitis and bacteremia.

### 3.3. Lenalidomide in the Setting of Infection

In our patient, a history of multiple prosthetic joints significantly elevated the risks associated with bacteremia due to her increased susceptibility to joint seeding. While most documented cases of *Pasteurella* bacteremia are in patients who suffered animal bites and scratches, there are documented cases in which the source of the infection was considered to be licking of an existing wound or kissing of the animal [3, 4, 13]. Of significant interest is the fact that the patient was taking lenalidomide at the time of hospitalization. In a meta-analysis conducted in 2017, the incidence of high-grade infection in patients with multiple myeloma taking lenalidomide was determined to be 14.32%, nearly twice the infection rate of the control group [15].

While a mechanism for the way in which lenalidomide might lead to immunodeficiency has not yet been established, a number of confounding factors leading to immunodeficiency in patients with multiple myeloma include advanced median age of patients, effects of the disease on B cells, T cells, and natural killer cells, as well as additional medical therapies used in the treatment of multiple myeloma [16].

### 3.4. Prosthetic vs. Native Joint Seeding

The presence of a prosthetic joint in and of itself is an established risk factor for infection [17]. Furthermore, in bacteremia patients, the microbial load required to seed and infect a joint is lower [17]. In a review of literature regarding the seeding of joints by *P. multocida* from 1985 to 2019 (Table 2), there were 30 cases of infection reported involving a knee joint, seven cases of a hip joint, two cases of shoulder joint (including our patient in this report), one case of ankle joint, and one case of wrist joint. Of these, 28 of 30 (93%) knee infections were prosthetic, 7 of 7 (100%) hip infections were prosthetic, 1 of 2 (50%) shoulder infections was prosthetic, 0 of 1 (0%) ankle infection was prosthetic, and 0 of 1 (0%) wrist infection was prosthetic.

The decision of whether to salvage or replace a prosthetic joint after seeding has occurred is a complicated one. In the review described above, 13 joints were debrided with antimicrobials and salvaged, while 29 joints were removed (Table 2). Cost and associated risks with repeated arthroscopic surgery and spacer placement have to be weighed against the risk of chronic infection that a more conservative debridement and salvage may bring. Of the 13 cases in which the joint was salvaged, one death occurred leading to the conclusion that salvage of the joint (and other septic joints in the general population) was a poor decision due to the risk of sepsis that goes with retainment of an infected foreign body [11].

### 3.5. Meningitis and Septic Arthritis in the Setting of *Pasteurella* Bacteremia

Review of 13 cases in which meningitis occurred in the setting of *P. multocida* infection from 1983 to 2018 was conducted to analyze the relationship between meningitis and bacteremia. 10 of 13 (77%) cases occurred in the setting of *Pasteurella* bacteremia, with the remaining 3 cases not documenting the presence (Table 3).

Based on the review of outcomes from 13 cases, 10 patients had resolution of infection, two patients had negative long-term neurological outcomes, and one patient died (in the setting of HIV infection). These results support consideration of lumbar puncture in patients presenting with evidence of joint infection in the setting of animal contact and immunocompromising illness due to the risk of meningitis.

### 3.6. Risk Factors and Potential Mechanism

While *Pasteurella* bacteremia in the absence of a bite or scratch is uncommon, most documented cases were in the presence of some form of immunodeficiency [3, 12]. A number of risk factors have been established including advanced age (mean age of 63), alcohol consumption, tobacco use, and chronic liver disease [61]. Our patient in this case report demonstrated both age and alcohol risk factors. Our patient denied any bites or scratches, and physical examination corroborated her story; however, there was an abrasion on her right wrist that she sustained from her prior fall. In support of the hypothesis that the licking of an already existing wound led to infection with *P. multocida*, the patient has multiple dogs and cats and reported that one cat has a tendency to lick people and objects. Cases in which licking was considered to be a likely mode of transmission have been documented [4, 62]. Additionally, one retrospective review noted that cases of *P. multocida* bacteremia where animal bites were not noted as the inciting event for infection were found to have poorer outcomes [63]. Given our patient's hospital course, we would hypothesize that her infection was related to licking of a preexisting wound, with underlying comorbidities portending a more aggressive course and potentially a worse prognosis.

### 3.7. Thrombocytopenia in the Setting of Infection

The patient was thrombocytopenic on initial labs taken in the emergency department. This resolved by day 3 of admission, and the patient had a normal platelet count for the duration of her hospital stay. A number of mechanisms have been postulated to account for thrombocytopenia in systemic infection including the formation of thrombocyte/neutrophil complexes and the temporary reduction of thrombopoiesis due to LPS [64, 65]. The role of platelets in infection has been reviewed at length by Dewitte et al. [66]. Given that this patient had a Gram-negative bacteremia, we suspect her temporary thrombocytopenia may have been due to a combination of thrombocyte/neutrophil aggregation and the effects of LPS on thrombopoiesis.

## 4. Conclusion

Given the relative abundance of cats and dogs as pets in the United States, it is prudent to remain aware of zoonotic pathogens. *P. multocida* is a microorganism with the capability of causing infection associated with significant morbidity and mortality. With pet ownership in the United States unlikely to decline in the near future, it is important to consider *P. multocida* in patients who have contact with pets and comorbid factors known to attenuate immunity. Physicians treating patients found to be infected with *P. multocida* should be aware of the potential for bacteremia, prosthetic joint infection, and meningitis, particularly in patients with advanced age and immunocompromised states as demonstrated by our patient.

## Figures and Tables

**Table 1:** Laboratory values from the patient's hospital admission.

Hospital day	Day 1	Day 2	Day 3	Day 4	Day 5	Day 6	Day 7	Day 8	Day 9	Day 10	Day 11	Day 20
WBC	9.9	7.1	14.1	14.1	11.5	10.7	9	10	10.4	8.3	8.7	6.0
RBC	2.8	2.76	2.9	3.06	2.65	3.01	3.05	2.92	2.81	2.7	2.81	2.69
Hemoglobin	9.6	9.1	9.7	9.9	8.8	9.8	9.9	9.7	9.3	8.6	9.3	8.5
Hematocrit	26.7	26.7	26.9	28	24.5	28.3	28.3	28.5	27.7	26.8	27.9	26.3
MCV	95.4	96.7	92.8	91.5	92.5	94	92.8	97.6	98.6	99.3	99.3	97.8
MCH	34.3	33	33.4	32.4	33.2	32.6	32.5	33.2	33.1	31.9	33.1	31.6
MCHC	36	34.1	36.1	35.4	35.9	34.6	35	34	33.6	32.1	33.3	232.3
RDW	13.9	14.3	14	14.4	14.5	14.6	14.1	14.5	13.9	13.7	13.2	13.6
Platelets	125	109	159	256	256	311	340	331	360	358	335	255
MPV	9.5	9.9	10	9.5	9.4	9.3	9	8.9	9.1	9	9	8.5
nRBC percentage	0	0	0	0	0	0	0	0	0	0	0	0
nRBC abs	0	0	0	0	0	0	0	0	0	0	0	0
Sodium	126	131	132	132	133	132	127	132	130	129	130	131
Potassium	4.3	2.9	309	3.1	3.4	3.7	3.9	4.5	4.5	4.1	4.2	3.6
Chloride	93	104	106	99	101	96	95	96	97	97	95	96
CO_2_	17	17	15	24	26	27	24	28	19	21	26	27
Anion gap	16	10	11	9	6	9	8	8	9	11	9	8
Glucose	93	80	90	104	99	99	93	90	78	78	88	104
BUN	21	14	13	7	7	7	8	12	12	12	11	6
Creatinine, serum	0.71	0.6	0.62	0.56	0.55	0.55	0.54	0.6	0.57	0.57	0.59	0.52
eGR non-African American	88	>90	>90	>90	>90	>90	>90	>90	>90	>90	>90	>90
Calcium	8.7	7.5	7.5	8.1	8	8.8	8.6	9.5	8.3	8.5	8.8	8.2
Bilirubin, total	1.6	1.0	1.2	1.2	1.0	1.0	1.0	0.8	0.6	06	0.4	0.3
Protein, total	6.8	5.2	5.2	5.6	5.1	6.0	6.1	6.3	5.8	6.0	6.2	5.3
Albumin	2.5	1.9	1.9	2.0	1.8	2.1	2.1	2.3	2.5	2.1	2.2	1.9
AST	213	206	105	49	25	22	24	22	24	19	20	12
ALT	57	76	56	42	24	19	17	15	14	11	10	7
Alkaline phosphatase	479	415	401	426	356	453	485	487	453	415	402	253
Magnesium		1.8	1.4	1.8	1.4	1.5	1.4	1.6	1.6	1.7	1.7	2.1
Ammonia												
Lactate	1.94	0.8										
CRP, inflammatory	237							133.3				
Procalcitonin	5.48											
CK, total	69	72										
Osmolality, measured										262		
ESR	104							58				

**Table 2:** Previous findings and outcomes from case reports in relation to the presence of septic arthritis in patients with *Pasteurella* bacteremia.

Reference	Site of infection	Animal exposure?	Comorbidities	Duration of therapy	Antibiotic resistance?	Bacteremia (y/n)?	Outcome (surgical intervention?)
Maurer et al. [18]	Knee, prosthetic	Yes	Rheumatoid arthritis	—	None	No	Cure, no intervention
Griffin and Barber [19]	Knee, prosthetic	Yes	Rheumatoid arthritis	—	None	No	Cure, no intervention
Sugarmen et al. [20]	Knee, prosthetic	Yes	Rheumatoid arthritis	—	None	No	Cure, replacement of prosthesis
Arvan and Goldberg [21]	Knee, prosthetic	Yes	Advanced age	—	None	No	Cure, surgical debridement
Spagnuolo [22]	Knee, prosthetic	Yes	Advanced age	—	None	No	Cure, surgical debridement
Gomez-Reino et al. [23]	Knee, prosthetic	Yes	Advanced age	—	None	No	Cure, replacement of prosthesis
Mellors and Schoen [24]	Bl knees, prosthetic	Yes	None	—	None	No	Cure, none
Orton and Fulcher [25]	Bl knees, prosthetic	Yes	Advanced age	—	None	No	Cure, replacement of prosthesis
Taillan et al. [26]	Knee, prosthetic	Yes	Advanced age	—	None	No	Cure, none
Braithwaite and Giddins [27]	Hip, prosthetic	Yes	Diabetes	—	None	No	Cure, replacement of prosthesis
Guion and Sculco [28]	Knee, prosthetic	Yes	Rheumatoid arthritis	—	None	No	Cure, replacement of prosthesis
Gabuzda and Barnett [29]	Knee, prosthetic	Yes	Advanced age	—	None	No	Cure, replacement of prosthesis
Antuña et al. [30]	Knee, prosthetic	Yes	Rheumatoid arthritis	—	None	No	Cure, surgical debridement
Takwale et al. [31]	Hip, prosthetic	Yes	Rheumatoid arthritis	—	None	No	Cure, replacement of prosthesis
Maradona et al. [32]	L knee, prosthetic	Yes	Advanced age, diabetes	3 weeks	None	No	Cure, surgical debridement
Chikwe et al. [33]	R hip, prosthetic	Yes	Advanced age	—	None	No	Cure, replacement of prosthesis
Ciampolini et al. [34]	Knee, prosthetic	Yes	Advanced age	6 weeks	None	No	Cure, replacement of prosthesis
Polzhoferet al. [35]	R knee, prosthetic	Yes	Advanced age	—	None	No	Cure, surgical debridement
Stiehl et al. [36]	Knee, prosthetic	Yes	Advanced age	—	None	No	Cure, replacement of prosthesis
Mehta and Mackie [37]	Hip, prosthetic	Yes	Advanced age, rheumatoid arthritis	—	None	No	Cure, replacement of prosthesis
Mehta and Mackie [37]	Hip, prosthetic	Yes	Rheumatoid arthritis	—	None	No	Cure, replacement of prosthesis
Heym et al. [38]	Knee, prosthetic	Yes	Advanced age	2 months	None	Yes	Cure, replacement of prosthesis
Kadakia and Langkamer [39]	Knee, prosthetic	Yes	Advanced age	10 weeks	None	No	Cure, surgical debridement
Heydeman et al. [40]	L knee, prosthetic	Yes	Advanced age	4 weeks	None	No	Cure, removal of prosthesis
Romano et al. [41]	L knee, prosthetic	Yes	Advanced age, rheumatoid arthritis	6 weeks	None	Yes	Cure, surgical debridement
Furgeson et al. [42]	L knee, prosthetic	Yes	Advanced age	8 weeks	None	No	Cure, surgical debridement
Ayoade and Todd. [17]	R knee, prosthetic	Yes	Advanced age, obesity, aortic stenosis	7 days	None	Yes	Death, surgical debridement
Arbefeville et al. [43]	R knee, prosthetic	Yes	Advanced age	6 weeks	None	yes	Cure, replacement of prosthesis
Zahirovic and Siddique [44]	L wrist, native	Yes	Diabetes	2 weeks	Yes (to erythromycin)	Yes	Cure, surgical debridement
Honnerat et al. [45]	Hip, prosthetic	Yes	Advanced age	8 months	None	No	Cure, surgical debridement
Honnerat et al. [45]	Knee, prosthetic	Yes	Advanced age, diabetes	8 months	None	No	Cure, replacement of prosthesis
Honnerat et al. [45]	Knee, prosthetic	Yes	Advanced age, diabetes	8 months	None	No	Cure, replacement of prosthesis
Honnerat et al. [45]	Knee, prosthetic	Yes	Advanced age, obesity	8 months	None	No	Cure, replacement of prosthesis
Honnerat et al. [45]	Knee, prosthetic	Yes	Advanced age	8 months	None	No	Cure, surgical debridement
Honnerat et al. [45]	Knee, prosthetic	Yes	Advanced age	8 months	None	No	Cure, replacement of prosthesis
Nitorslawski et al. [46]	Knee, native; shoulder, native	Yes	Advanced age	6 weeks	None	No	Cure, surgical debridement
Fayyaz [47]	L hip, prosthetic	No	COPD, alcohol use	6 weeks	None	Yes	Cure, replacement of prosthesis
Katechakis et al. [48]	Ankle and knee, native	Yes	None	20 days	None	Yes	Cure, below the knee amputation
			Alcohol use,				

**Table 3:** Previous findings and outcomes from case reports in relation to the presence of meningitis in patients with *Pasteurella* bacteremia.

Reference	Year	Presenting compliant	Site of infection	Animal exposure?	Comorbidities	Antibiotic resistance?	Bacteremia (y/n)?	Outcome
Nitoslawski et al. [46]	2018	Fever	CNS	Yes	Diabetes mellitus	No	Yes	Cure
Larné et al. [49]	2019	Fever	CNS	Yes		No	Yes	Cure
Clarke et al. [2]	2017	Fever	CNS	Yes		No	Yes	Cure, residual hearing loss
Yamaguchi et al. [50]	2014	Fever	CNS	Yes	None	No	Yes	Cure
Spadafora et al. [51]	2011	Status epilepticus	CNS	Yes		No	No	Cure
Soloaga et al. [52]	2008	Fever	CNS	Yes		No	Yes	Cure
Hirsh et al. [53]	2004	Fever	CNS	Yes	None	No	No	Cure
Green et al. [54]	2002		CNS	Yes		No	Yes	Cure
Layton [55]	1999	Fever	CNS	Yes		No	Yes	Cure
Guerin et al. [56]	1994		CNS	Yes	HIV+	No	Yes	Death
Kumar et al. [57]	1990		CNS	Yes		No	Yes	Cure
Levy et al. [58]	1989	Fever	CNS	Yes	None	No	Yes	Cure, residual neurological deficit
Permezel et el. [59]	1984	Fever	CNS	Yes	Recent sinus surgery	No	No	Cure
Bruun and Friis-Møller [60]	1983	Fever	CNS	Yes	Chronic om	No	No	Cure

## Abbreviations

g/dL: Grams per deciliter
mMol/L: Millimoles per liter
IU/L: International units per liter
MRI: Magnetic resonance imaging
CT: Computed tomography
LPS: Lipopolysaccharide.
